# Actor and partner effects of parenting stress and co-parenting on marital conflict among parents of children with atopic dermatitis

**DOI:** 10.1186/s12887-020-02035-7

**Published:** 2020-03-30

**Authors:** Jeong Won Han, Hanna Lee

**Affiliations:** 1 College of Nursing Science, Kyung Hee University, 24 Kyungheedae-ro, Dongdaemun-gu, Seoul Republic of Korea; 2 Department of Nursing, Gangneung-Wonju National University, 150, Namwon-ro, Heungeop-myeon, Wonju-si, Gangwon-do Republic of Korea

**Keywords:** Child, Conflict, Dermatitis, Parents, Stress

## Abstract

**Background:**

It is important for healthcare providers to pay attention to parents’ rearing style and children’s physical symptoms to promote a healthy quality of life among children with atopic dermatitis. We aimed to identify effects of parenting stress and co-parenting on marital conflict among parents of children with atopic dermatitis.

**Methods:**

Participants were 161 fathers and 161 mothers raising seven-year-old children treated for atopic dermatitis. To confirm the effects of parenting stress and co-parenting on marital conflict, the “actor-partner interdependence mediation model” was used. To verify goodness-of-fit, maximum likelihood method was used, and a confirmatory factor analysis was conducted to confirm the validity of latent variables for model analysis.

**Results:**

Fathers’ parenting stress had actor (β = −.46, *p* < .001) and partner (β = −.22, *p* < .001) effects on co-parenting, and mothers’ parenting stress had actor (β = −.20, *p* < .001) and partner (β = −.36, *p* < .001) effects on co-parenting. Fathers’ parenting stress only had an actor effect on marital conflict (β = .32, *p* < .001). Father’s co-parenting had actor (β = −.29, *p* < .001) and partner (β = −.22, *p* < .001) effects on marital conflict, and mothers’ co-parenting had actor (β = −.39, *p* < .001) and partner (β = −.19, *p* < .001) effects on marital conflict. There were significant differences between the two groups concerning three path coefficients: fathers’ parenting stress affected fathers’ marital conflict, fathers’ co-parenting affected fathers’ marital conflict, and mothers’ co-parenting affected fathers’ marital conflict.

**Conclusions:**

It is vital for healthcare providers to seek ways to reduce the marital conflicts of parents of children with atopic dermatitis, including further examination of the role of co-parenting, to address children’s physical symptoms and promote their health. Our findings inform management and intervention programs for the families of children with atopic dermatitis.

## Background

Atopic dermatitis (AD) is the most prevalent sustained chronic inflammation and pruritic skin disease, affecting many infants and children in industrialized countries [1]. In Korea, according to the Ministry of Health and Welfare’s Korea Health Statistics, the prevalence of AD is steadily increasing: it was 2.4% in 2007, 3.3% in 2010, and 3.8% in 2015 [2]. According to a survey on the prevalence of allergic diseases in Korea among 933,000 patients, AD had a proportionate morbidity rate of 48.6% in patients aged younger than 12 years, followed by 12.7% in those aged 13–19 years and 11.8% in those in their 20s; this indicates that the patients are more commonly children and adolescents than in other age groups [3]. In particular, the symptoms of AD peak between four and six years [4], and they often develop into allergic rhinitis or asthma; therefore, continuous management is needed [5].

AD not only causes various physical issues, it also leads to psychological problems; patients experience frequent skin damage and sleep disorders owing to extreme pruritus [6] and show depression, anxiety, attention deficit, tiredness, irritable mood, and aggressive behavior [7]. Parents of children with AD must strictly manage AD daily, such as consistent skin moisturization, food preparation, and environmental management, as well as general childcare for each stage of children’s growth; therefore, in addition to the pain experienced by the child, the degree of parenting stress is relatively higher than with other diseases [8]. A study concerning mothers of children with eczema in Sydney, Australia [9] showed that the mothers experienced relatively higher levels of stress than did the mothers of children with other chronic conditions. As such, parents of children with AD experience psychological crises—such as guilt, hopelessness, frustration, and exhaustion—which negatively affect family functioning [10].

Recent research has reported that the relationship between parents and children is closely related to children’s growth and development [11]. From an ecological perspective, parents and children are members of the family system [12]; therefore, the are connected as a unit. Consequently, it is important to approach children’s health problems by understanding parents’ concerns and the familial status. In particular, parents’ emotions are affected by spouses’ stress levels [13]. This crossover effect in the family system [14] indicates how interactions among family members affect the emotions of other members [15]. Moreover, parents’ emotions can affect children’s relationships, growth, and development; i.e., spillover effects [14, 15].

Therefore, the health of a child with AD is not just the child’s problem; rather, it is associated with parental variables. It is thus important for society and medical practitioners to understand the situational factors of parents who raise children with AD. Particularly, when a child has an atopic disease, parents must play the role of healthcare providers as well as their ordinary parenting roles [13]. Owing to the characteristics of atopic diseases, long-term management is a factor that increases parents’ stress. Prior studies have shown that mothers, who are more commonly involved in child rearing than are fathers, feel guilty about children’s symptoms [16] and exhibit greater parenting stress than do fathers. However, as fathers’ role in child rearing is increasing, so too is their parenting stress [17, 18]. Further, mothers who are professionals tend to have relatively more parenting stress than do full-time homemakers because they have to balance work and family [18]. Since modern society currently demands equal parenting responsibilities from fathers and mothers, it is necessary to determine the role of parenting stress at the individual level and concerning the couple as a unit [19].

Parenting stress is distinct from the general stress experienced in daily or social life. When parents recognize that they lack the available personal and social support in the process of fulfilling the roles required by society, they experience stress [20]. This parenting stress causes parents to give up their roles or to become passive, and it often makes them avoid child-rearing responsibilities or generates marital conflicts [13]. Consequently, co-parenting becomes an important factor that plays a mediating role in parenting and marital relationships [17]. Co-parenting implies that both parents are aware of their roles and participate in parenting [21]. It means not only sharing responsibility for raising children, but also cooperating and supporting each other in the parenting process. Additionally, since the concept of co-parenting has been extended to sharing the beliefs, values, hopes, expectations, and cultures of individuals in modern society, co-parenting is also affected by the family system [22]. Since parenting stress can increase when spousal social support is low, effective co-parenting can reduce parenting stress and marital conflicts [17]. The co-parenting model suggested by Feinberg [11] also reported that co-parenting could reduce parenting stress and promote children’s (and parents’) adaptation.

Consequently, to promote the health of children with AD, maintaining familial function including decreasing marital conflict and fostering co-parenting is critical. However, co-parenting can also differ depending on parents’ personal characteristics [23]. Particularly, it may vary according to the diverse roles of mothers. Therefore, it is vital to examine mothers’ roles to determine effective approaches to manage the symptoms of children with AD.

Previous studies concerning children with AD examined the effect of parents’ self-efficacy and marital satisfaction on children’s behavior [24], the effect of mothers’ self-efficacy on family management [25], and sleep disorders in parents raising children with AD [26]; however, limited research has comprehensively approached the relationships between children with AD and their parents. In particular, considering that children are highly dependent on parents owing to the nature of AD, studies that investigate multiple parental variables concurrently are necessary for the management of children with AD.

Because parents are in an interdependent relationship, the actor-partner interdependence mediation model (APIMeM) suggested by Kenny and colleague [27] is recommended to analyze the interrelationships between parent-related variables. This study applied the APIMeM model to identify the effects of parenting stress and co-parenting on marital conflict among parents of children with AD. For couples’ data, the mutual dynamics of the couple were not reviewed when using individual data. Even if data were collected from both members of the couple, analyzing such interdependent data individually as independent data violates the main hypothesis of reasoning statistics, resulting in low calculation of standard error and a possibility of committing a Type 1 error. Thus, such interdependent couple data must be analyzed by applying APIMeM [27]. In particular, marital conflict is a result of the interaction between parents; therefore, it is necessary to determine the effects of parenting stress and co-parenting instead of analyzing mothers and fathers individually.

### Aim

In sum, we examined the effects of parenting stress and co-parenting on marital conflict, the actor and partner effects of parental variables, and the control effect of mothers’ employment to provide basic data for the development of an AD family-management program.

## Methods

### Design

**Fig. 1:**
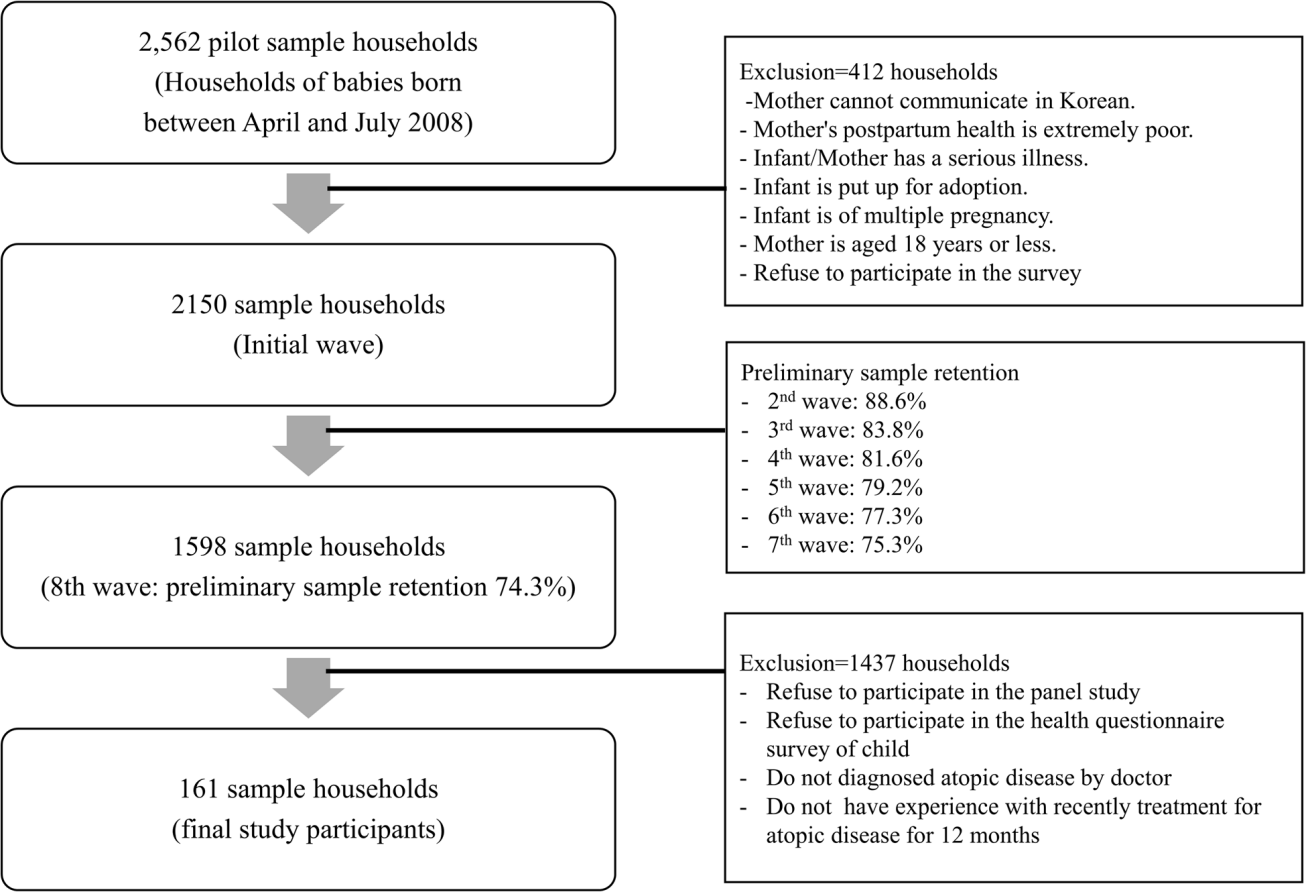
The process on selection of subjects

This was a cross-sectional study that utilized the 8th Panel Study on Korean Children [Fig. 1].

### Participants

We included parents and their children who participated in the 8th Panel Study on Korean Children (2015). The Panel Study was a review of the newborns born in 2008, their mothers, and the community environment (the date are publicly available). The Panel Study, conducted by the Korea Institute of Child Care and Education, included all households of newborns (excluding those who refused to participate) born between April and July 2008 from surveyed medical institutes with more than 500 or more annual births per year. The exclusion criteria were mothers who could not communicate in Korean, mothers with poor health after giving birth, newborns with serious diseases, mothers with serious diseases, newborns awaiting adoption, multiple births, and mothers aged ≤18 years.

The Panel Study recruited a pilot sample of 2563 households, from which 2150 households were selected as the final sample. Stratified multistage sampling was applied: the first stage included selecting medical institutes where childbirth occurs, the second stage included extracting households with newborns born in selected medical institutes as a pilot sample, and the third stage included establishing a sample from the pilot sample with households who wished to participate in the panel. The sample retention rate proposed by the Panel Study’s research team for the validity of this study sample was 74.3%.

**Fig. 2:**
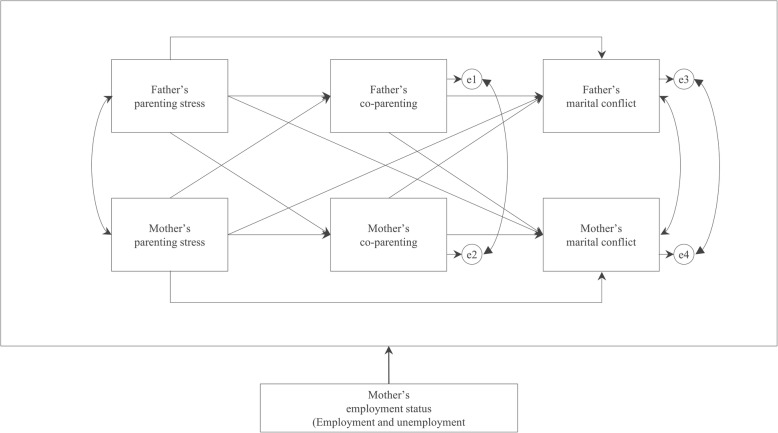
Hypothetical model of the study

In the current study, among all children who participated in the Panel Study and health questionnaire survey, 161 fathers and 161 mothers raising seven-year-old children, treated for AD within the last year, were selected as the final study participants [Fig. 2]. The Korean Children’s Panel Survey requested the Asan Medical Center to develop a questionnaire related to children’s health and verified the presence of atopy from the parents of the children through trained surveyors. The presence of children with AD was confirmed by using questions such as, “Has your child been diagnosed with AD by a doctor?,” “When was your child first diagnosed with atopy?,” and “Has your child been treated owing to AD (also known as “congenital fever” or “eczema”)?”

In the structural equation model, the minimum recommendation for the sample size is 10 times the free parameter, and the ideal size is 150–400 participants [28]; therefore, 161 participants constituted a sufficient sample size to analyze actor and partner effects using the structural equation model.

### Measurements

#### Parenting stress

For the parenting stress survey, “burden and distress from carrying out parents’ role,” among the subfactors of the parenting stress scale developed by Kim and Kang [29], was extracted by the Panel Study’s research team, and a tool with 11 questions—confirmed through a preliminary survey from 2007—was used. Eleven questions were based on a five-point scale, and higher scores signified higher parenting stress. Concerning tool reliability, Cronbach’s alpha was .86 in a previous study [29], and .88 (fathers) and .90 (mothers) in this study.

The confirmatory factor analysis revealed that the goodness-of-fit of fathers’ parenting stress model was χ^2^ = 26.24, df = 24, goodness-of-fit index (GFI) = .93, adjusted GFI (AGFI) = .90, normed fit index (NFI) = .92, comparative fit index (CFI) = .94, root mean squared error of approximation (RMSEA) = .02. The goodness-of-fit of mothers’ parenting stress model was χ^2^ = 70.49, df = 24, GFI = .93, AGFI = .90, NFI = .92, CFI = .94, RMSEA = .05.

#### Co-parenting

Co-parenting is a conceptual term that refers to the ways that parents and/or parental figures relate to each other in the role of a parent. For the co-parenting survey, the measurement tool developed by Mchale [30] was translated by the Panel Study’s team, and 16 questions (four subcategories: family unity, discipline, criticism, conflict) were selected. Questions were answered with a seven-point scale. Higher scores signified a higher level of co-parenting. In Mchale’s study [30], Cronbach’s alphas ranged .59–.82; in this study, Cronbach’s alphas .88 (fathers) and .86 (mothers). The confirmatory factor analysis revealed that the goodness-of-fit of the fathers’ co-parenting model was χ^2^ = 34.23, df = 21, GFI = 95, AGFI = .91, NFI = .94, CFI = .96, RMSEA = .05. The goodness-of-fit of mothers’ co-parenting model was χ^2^ = 31.13, df = 21, GFI = .97, AGFI = .92, NFI = .97, CFI = .98, RMSEA = .06.

#### Marital conflict

For marital conflict, the measurement tool developed by Markman et al., [31] was translated and revised by the Panel Study’s research team. It comprised eight questions that were answered using a five-point scale. Cronbach’s alphas for fathers and mothers were .91 and .93, respectively. The confirmatory factor analysis revealed that the goodness-of-fit of fathers’ marital conflict model was χ^2^ = 49.55, df = 20, GFI = .93, AGFI = .90, NFI = .94, CFI = .96, RMSEA = .03. The goodness-of-fit of mothers’ marital conflict model was χ^2^ = 56.32, df = 20, GFI = .92, AGFI = .90, NFI = .95, CFI = .97, RMSEA = .04.

### Ethical considerations

The 8th Panel Study on Korean Children was approved by the institutional review board of the Korea Institute of Child Care and Education (no. KICCEIRB-2015-03). The current work was also conducted after review by the Institutional Review Board of C University (no. 1040271-201811-HR-030).

### Data collection and analysis

The data were obtained from the Panel Study on Korean Children’s website (http://panel.kicce.re.kr/kor/publication/02.jsp). For data use, the study protocol was submitted to the Panel Study’s research team and reviewed. After obtaining approval, the corresponding data were downloaded. The collected data were analyzed using IBM SPSS Statistics for Windows, Version 22.0 (IBM Data solution, Seoul, Korea) and IBM SPSS AMOS, Version 20.0 programs (IBM Data solution, Seoul, Korea). Descriptive statistics were used for participants’ general characteristics and descriptive statistics of the measurement variables. Skewness and kurtosis of the measurement variables were verified for the normality of the data. For each measurement variable, the absolute value of skewness (− 0.65 to 0.81) did not exceed 2, and the absolute value of the kurtosis (− 0.17 to 1.15) did not exceed 4. AMOS was used to confirm multivariate normality. In this study, the univariate normality of each measurement variable satisfied the normal distribution condition by showing the absolute value of the skewness and the kurtosis ranging less than 2; however, multivariate normality was not satisfied at the significance level of .05 with multivariate index = 4.10 and CR = 6.10. If multivariate normality is not satisfied, there may be a problem of upward biasing the threshold when estimating the parameters. However, even if the multivariate normality is not assumed, it is reported that the estimated parameter is reliable when using the maximum likelihood method and when the sample size is ≥120. Therefore, the model was estimated without converting the data.

In addition, the correlations and multicollinearity of each construct and the measurement variables were confirmed by Pearson’s correlation coefficient, and the reliability of the tool was confirmed by Cronbach’s alpha coefficient. To confirm the actor and partner effects of parenting stress and co-parenting on marital conflict, the AMOS structural equation model was used. Furthermore, measurement invariance was conducted to confirm the homogeneity of fathers’ and mothers’ data within one measurement tool. To verify this, four competing models were compared. The first model was the baseline model, the second constrained the factor loading, the third constrained the covariance of the error, and the fourth constrained the factor loading and covariance of the error.

To verify the goodness-of-fit of our model, maximum likelihood method was used, and a confirmatory factor analysis was conducted to confirm the validity of latent variables for model analysis. For the goodness-of-fit of the model, the absolute fit indices of χ^2^, χ^2^/df, RMSEA, SRMR, GFI, AGFI, CFI, NFI and the Tucker-Lewis Index (TLI) were used. Direct effects, indirect effects, and total effect significance were confirmed using bootstrapping. To test structural model invariance across groups, an analysis technique that examines the difference in path coefficients between measurement models was used to compare the critical ratios of the free and constrained models.

## Results

### Participants’ general characteristics

Concerning participants’ residences, 62 lived in large cities (38.5%), 69 lived in towns (42.9%), and 30 lived in small and medium-size cities. The mean age of the fathers was 40.5 ± 3.98 years, while the mean age of the mothers was 37.9 ± 3.73 years. Concerning education, 81 fathers (50.3%) and 70 mothers (43.5%) had a bachelor’s degree. As for occupation, 69 (42.9%) of the fathers were managers/office workers. For mothers, 66 (41.0%) were employed, and 95 (59.0%) were unemployed. Among the working mothers, 48 (29.8%) were managers/office workers. The mean household income was 471.5 ± 238.63 million won. Concerning the sex of the children, 93 (57.8%) were boys and 68 (42.2%) were girls. Concerning time of AD diagnosis of, 49 (30.4%) were diagnosed within 12 months of birth, 53 (32.9%) between 15- and 35-months-old, 32 (19.9%) between 3- and 4-years-old, and 27 (16.7%) after 5-years-old.

### Descriptive statistics of measurement variables

The mean parenting stress score of the fathers was 1.9 points (range = 1.0–4.0), and that of the mothers was 2.3 points (range = 1–4.7). The mean co-parenting score of the fathers was 5.2 points (range = 3–7), and that of the mothers was 5.4 points (range = 2.5–7). The mean marital conflict score of the fathers was 1.9 points (range = 1–4.3), and that of the mothers was 2.1 points (range = 1–4.4) [Table 1].

**Table 1:** Correlation of the variables

							(*N* = 161)	
Variables	M ± SD	1	2	3	4	5	6	
1: Parenting stress (Father)	1.91 ± 0.59	1						
2: Parenting stress (Mother)	2.37 ± 0.70	.33^***^	1					
3: Co-parenting (Father)	5.24 ± 0.97	−.52^***^	−.35^***^	1				
4: Co-parenting (Mother)	5.40 ± 0.99	−.34^***^	−.43^***^	.50^***^	1			
5: Marital conflict (Father)	1.98 ± 0.65	.52^***^	.23^**^	−.53^***^	−.41^*^	1		
6: Marital conflict (Mother)	2.16 ± 0.81	.32^***^	.38^***^	−.47^*^	−.56^***^	.56^***^	1	

*M* mean, *SD* standard deviation, *p < 0.05; **p < 0.01; ***p < 0.001

### Correlation between measurement variables

Each measurement variable was significantly correlated with each other (*p* < .05), and the absolute value of the correlation between the variables did not exceed .8—confirming that there was no problem of multicollinearity [Table 1].

### Verification of measurement invariance of measurement variables

In this study, the results of χ^2^ and TLI, CFI, RMSEA, which are not sensitive to the number of cases, confirmed measurement invariance [Table 2].

**Table 2:** Verification of measurement invariance of measurement variables

Model		χ^2^	df	TLI	CFI	RMSEA
Parenting stress						
Model 1	Unconstrained model	233.59	76	.88	.92	.06
Model 2	Measurement weights constrain	268.90	88	.89	.90	.06
Model 3	Measurement residual constrain	217.60	69	.87	.92	.07
Model 4	Measurement weights and residual constrain	249.18	81	.88	.91	.06
Co-parenting						
Model 1	Unconstrained model	229.32	76	.88	.92	.05
Model 2	Measurement weights constrain	263.54	88	.89	.91	.05
Model 3	Measurement residual constrain	190.24	69	.91	.93	.04
Model 4	Measurement weights and residual constrain	242.21	81	.90	.92	.05
Marital conflict						
Model 1	Unconstrained model	216.30	103	.93	.94	.04
Model 2	Measurement weights constrain	323.95	117	.89	.89	.05
Model 3	Measurement residual constrain	188.95	95	.94	.95	.03
Model 4	Measurement weights and residual constrain	291.82	109	.90	.91	.05

*df* degrees of freedom *TLI* Tucker-Lewis Index, *CFI* Comparative Fit Index, *RMSEA* Root Mean Squared Error of Approximation

### Actor and partner effect of parenting stress and co-parenting on marital conflict

Our hypothetical model test revealed appropriate goodness-of-fit (χ^2^ = 15.59, df = 10, RMSEA = .02, SRMR = .04, GFI = .95, AGFI = .94, CFI = .97, NFI = .97, TLI = .96). Nine out of 12 hypotheses were selected [Table 3]. Fathers’ parenting stress had an actor effect (β = −.46, *p* < .001) on co-parenting and a partner effect (β = −.22, *p* < .001) on mothers’ co-parenting, and mothers’ parenting stress had an actor effect (β = −.36, *p* < .001) on mothers’ co-parenting and a partner effect (β = −.20, *p* < .001) on fathers’ co-parenting. Fathers’ parenting stress only had an actor effect (β = .32, *p* < .001) on fathers’ marital conflict. Fathers’ co-parenting had an actor effect (β = −.29, *p* < .001) on fathers’ marital conflict and a partner effect (β = −.22, *p* < .001) on mothers’ marital conflict, and mothers’ co-parenting had a partner effect (β = −.19, *p* < .001) on fathers’ marital conflict and an actor effect (β = −.39, *p* < .001) on mothers’ marital conflict [Table 3]. In addition, fathers’ parenting stress (β = .17, *p* = .004) had an indirect effect on fathers’ marital conflict, and fathers’ parenting stress had an indirect effect on mothers’ marital conflict (β = .04, *p* = .005); however, the total effect (β = .16, *p* = .269) of fathers’ parenting stress on mothers’ marital conflict was non-significant. Mothers’ parenting stress had an indirect effect on fathers’ (β = .07, *p* = .005) and mothers’ (β = .18, *p* = .003) marital conflict.

**Table 3:** Actor and partner effect of parenting stress and co-parenting on marital conflict

Independent variables		Dependent variables	β	B	S.E	C.R	*p*	*Direct effect*		*Indirect effect*		*Total effect*	
								β	*p*	β	*p*	β	*p*
Parenting stress (f)	➔	Co-parenting (f)	−.46	−.74	.11	−6.69	<.001	−.46	<.001	–	–	−.46	<.001
Parenting stress (f)	➔	Co-parenting (m)	−.22	−.37	.12	−3.12	<.001	−.22	<.001	–	–	−.22	<.001
Parenting stress (m)	➔	Co-parenting (f)	−.20	−.27	.09	−2.88	<.001	−.20	<.001	–	–	−.20	<.001
Parenting stress (m)	➔	Co-parenting (m)	−.36	−.50	.10	−4.94	<.001	−.36	<.001	–	–	−.36	<.001
Parenting stress (f)	➔	Marital conflict (f)	.32	−.32	.08	4.40	<.001	.32	<.001	.17	.004	.49	.023
Parenting stress (f)	➔	Marital conflict (m)	.12	.14	.10	0.87	.699	.12	.699	.04	.005	.16	.269
Parenting stress (m)	➔	Marital conflict (f)	.15	.15	.06	0.57	.449	.15	.449	.07	.005	.22	.007
Parenting stress (m)	➔	Marital conflict (m)	.13	.15	.08	1.82	.068	.13	.068	.18	.003	.31	.013
Co-parenting (f)	➔	Marital conflict (f)	−.29	−.20	.05	−3.64	<.001	−.29	<.001	–	–	−.29	<.001
Co-parenting (f)	➔	Marital conflict (m)	−.22	−.19	.07	−2.75	<.001	−.22	<.001	–	–	−.22	<.001
Co-parenting (m)	➔	Marital conflict (f)	−.19	−.12	.05	−2.40	<.001	−.19	<.001	–	–	−.19	<.001
Co-parenting (m)	➔	Marital conflict (m)	−.39	−.31	.06	−5.70	<.001	−.39	<.001	–	–	−.39	<.001

*f* father, *m* mother, *SE* Standard error, *C.R* Critical ratio

### Multiple group analysis of parenting stress, co-parenting, and marital conflict between unemployed and employed mothers

To confirm significant differences between the intergroup path coefficients, the critical ratio for difference of free and constrained models between the 12 paths in the study model was confirmed. There were significant differences between the two groups concerning the following path coefficients: fathers’ parenting stress affected fathers’ marital conflict (critical ratio for difference = − 2.408), fathers’ co-parenting affected fathers’ marital conflict (critical ratio for difference = 2.753), and mothers’ co-parenting affected fathers’ marital conflict (critical ratio for difference = 2.952) [Table 4].

**Table 4:** Multiple group analysis on parenting stress, co-parenting and marital conflict between unemployed and employed mothers

Independent variables		Dependent variables	*Employed*		*Unemployed*		*Critical ratios of difference*
			β	*p*	β	*p*	
Parenting stress (f)	➔	Co-parenting (f)	−.46	<.001	−.46	<.001	−0.035
Parenting stress (f)	➔	Co-parenting (m)	−.18	.102	−.30	.001	0.146.
Parenting stress (m)	➔	Co-parenting (f)	−.22	.033	−.18	.044	−0.484
Parenting stress (m)	➔	Co-parenting (m)	−.37	<.001	−.35	<.001	0.342
Parenting stress (f)	➔	Marital conflict (f)	.41	<.001	.25	.012	−2.408
Parenting stress (f)	➔	Marital conflict (m)	.17	.129	.19	.342	0.598
Parenting stress (m)	➔	Marital conflict (f)	.18	.429	.10	.976	−1.775
Parenting stress (m)	➔	Marital conflict (m)	.14	.738	.21	.019	1.325
Co-parenting (f)	➔	Marital conflict (f)	−.41	<.001	−.18	.103	2.753
Co-parenting (f)	➔	Marital conflict (m)	−.30	.015	−.15	<.001	−1.625
Co-parenting (m)	➔	Marital conflict (f)	−.14	.657	−.29	.007	2.952
Co-parenting (m)	➔	Marital conflict (m)	−.31	.007	−.45	.137	1.274

## Discussion

This study aimed to identify the actor and partner effects of parenting stress and co-parenting on marital conflicts in parents raising children with AD and to further discuss the differences between groups based on mothers’ employment. First, the parenting stress of the fathers and mothers of children with AD had actor and partner effects on both parents’ co-parenting. These results are similar to the findings of May and colleagues [32] and Feinberg [11], who reported parenting stress to affect co-parenting based on the co-parenting model conducted in parents of children with autism.

Such results show that co-parenting is a process in which couples discuss the principles of child-rearing, share the burden of child-rearing, and cooperate with each other [33]. When mothers and fathers support each other, their parental confidence increases; however, conflict between spouses results in stress and decreased parental motivation [30]. Therefore, considering that the parents of children with AD have a higher level of parenting stress than do the parents of children with other chronic diseases, medical professionals should intervene to reduce parental stress and foster familial stability.

Second, marital conflicts perceived by the fathers of children with AD were influenced by the actor effect of fathers’ parenting stress and co-parenting and partner effect of mothers’ co-parenting. As reported in a previous study on marital conflict and parenting [34], marital conflicts increase when a couple perceives that they cannot get help and cooperation from their spouses while raising their children; specifically, the degree of fathers’ perceived marital conflict increases if the father of the child with AD believes that he did not get much help from the mother for parenting. Previous studies also noted that marital conflict is associated with children’s internal and external problem behaviors [34, 35], and it affects the restoration of health in children with AD; therefore, there is a need for medical professionals to address the perceived marital conflict of fathers of children with AD, and it is necessary to examine mothers’ participation in parenting and attitudes toward co-parenting to reduce the degree of marital conflict experienced by fathers.

Third, the marital conflict perceived by the mothers of children with AD was affected by the actor effect of mothers’ co-parenting and the partner effect of fathers’ co-parenting. Such results are similar to the results by Feinberg [11], who reported that co-parenting had a positive effect on the adaptation between husbands and wives. For parents, the marital relationship is closely related to the process of raising children. One study noted that children are more likely to be affected by their father’s emotional and behavioral status than their mother’s, which suggests the importance of fathers’ co-parenting in raising children with AD [35]. In addition, mothers could become dependent on fathers during child-rearing; during this process, mothers tend to underestimate the quality of their marital relationship if they perceive fathers’ level of parental involvement to be low. Therefore, to lower the degree of marital conflict perceived by the mothers of children with AD, it is necessary to confirm fathers’ attitudes toward and role in co-parenting in addition to fostering mothers’ attitudes toward co-parenting.

Fourth, parenting stress had an indirect effect on the marital conflict perceived by fathers, while mothers’ parenting stress had an indirect effect on the marital conflict perceived by mothers. Mothers are usually the primary caregiver of children with AD, and, because they feel great burden [9], active intervention for these women could be an important factor in reducing marital conflict.

In addition, in the path analysis, according to mothers’ employment and unemployment, there were significant differences among the groups concerning fathers’ parenting stress to fathers’ marital conflict, fathers’ co-parenting to fathers’ marital conflict, and mothers’ co-parenting to fathers’ marital conflict. Such results indicate that, when the mother is employed, the father’s parenting role is relatively high, which affects perceived co-parenting and thus marital conflict. Therefore, there is a need a distinctive intervention plan that addresses fathers’ parenting stress and co-parenting among parents of children with AD when mothers are employed. Further, if mothers are homemakers, the key factor that affects fathers’ marital conflict may be mothers’ co-parenting; thus, intervention plans should be revised accordingly.

## Conclusion

This study had some limitations. The Korean Children’s Panel Survey did not evaluate the severity of children’s AD, which could have influenced parents’ stress. Therefore, future studies need to include the severity of children with AD. A follow-up study is suggested to develop a family management program for children with AD considering actor and partner effects of parenting stress, co-parenting, and marital conflicts and to further verify the effects. In addition, parenting stress may appear distinctively according to the severity of symptoms in children with AD; therefore, it is necessary to determine these relationships. Lastly, follow-up studies are needed to identify various factors to alleviate stress.

In sum, it is vital for healthcare providers to seek ways to reduce the marital conflicts between parents of children with AD to promote familial stability. It is also critical to confirm the attitude and magnitude of parents’ co-parenting as a method to reduce marital conflict in said parents. Lastly, effective intervention programs should be devised for families of children with AD.

## Data Availability

Not applicable.

## Abbreviations

AD: Atopic dermatitis
CFI: Comparative fit index
CR: Critical ratio
df: Degrees of freedom
GFI: Goodness-of-fit index
NFI: Normed fit index
RMSEA: Root mean squared error of approximation
SRMR: Standardized root mean square residual
TLI: Tucker-Lewis Index
